# DEMENTED OLDER ADULT’S SALIVARY CORTISOL LEVELS AND PHYSICAL STRESS INDEX AS STRESS INDICATOR

**DOI:** 10.1093/geroni/igz038.3205

**Published:** 2019-11-08

**Authors:** Eunji Kwon, Eunhee Cho

**Affiliations:** 1 Yonsei university, Seoul, Korea, Republic of; 2 Professor, College of Nursing, Yonsei University, Seoul, Korea, Republic of

## Abstract

Demented older adults experience many internal and external stress inducers that are thought to be a source of behavioral and psychological symptoms of dementia(BPSD). The purpose of this study was to compare the stress index among older adults through salivary cortisol levels and physical stress index. This study was cross-sectional design, including 139 participants who recruited until May of this year(104 demented older adults who visited hospital outpatient neurology and 35 non-demented older adults as control group). The physical stress index was measured by heart rate variability and salivary cortisol levels(4 samples/day, 1 days). Salivary cortisol levels were measured at four times after wake up, after breakfast, before dinner and after dinner. The data were analyzed using independent t-test and generalized estimating equations. In salivary cortisol levels measured after wake up, the demented older adults reported about 1.5 times higher than non-demented older adults(p=.042). And the salivary cortisol levels measured after breakfast were about 2.3 times higher in the demented older adults than in control groups(p=.002). Accordingly, the results can be concluded that demented older adults have higher stress levels than control groups in the morning. Also the physical stress index through heart rate variability(HRV) in the demented older adults(6.30±0.65) had higher than control groups(6.00±0.55, t=2.45, p=.016). There are significant differences in salivary cortisol levels and physical stress index between demented older adults and control groups. As stress inducers affects BPSD for the demented older adults, nursing intervention should be tailored to proper way based on their stress inducers.

